# Solvent assisted evolution and growth mechanism of zero to three dimensional ZnO nanostructures for dye sensitized solar cell applications

**DOI:** 10.1038/s41598-021-85701-9

**Published:** 2021-03-17

**Authors:** M. Ramya, T. K. Nideep, V. P. N. Nampoori, M. Kailasnath

**Affiliations:** International School of Photonics, Cochin University of Science and Technology, Kochi, India

**Keywords:** Chemistry, Energy science and technology, Materials science, Nanoscience and technology

## Abstract

We report the structural engineering of ZnO nanostructures by a consistent solution method using distinct solvents such as ethylene glycol, 1-butanol, acetic acid and water. The growth kinetics are found to depend strongly on the physicochemical properties of the solvent and zeta potential of the colloidal solution. Furthermore, the resulting nanostructures as a photoanode material, displayed a prominent structure dependent property in determining the efficiency of dye-sensitized solar cells (DSSCs). The fabricated solar cell with ZnO nanostructures based photoanode exhibited improved conversion efficiency. Moreover, the nanoflower based DSSCs showed a higher conversion efficiency of 4.1% compared to the other structures. The excellent performance of ZnO nanoflower is attributed to its better light-harvesting ability and increased resistance to charge-recombination. Therefore ZnO nanostructures can be a promising alternative for TiO_2_ in DSSCs. These findings provide new insight into the simple, low cost and consistent synthetic strategies for ZnO nanostructures and its outstanding performance as a photoanode material in DSSCs.

## Introduction

A reliable, cost-effective and everlasting renewable energy source like solar energy is the solution for the next generation energy crisis. The current solar energy market is dominated by the silicon-based p–n junction photovoltaic devices because of their maturity, performance and reliability. Since the low-cost investments and fabrication are the key desired features, researchers everywhere are trying to come up with newer and more economical ways of generating energy through solar power^1,2^. Dye-sensitized solar cells (DSSCs) have attracted much attention in recent years because of their good photovoltaic performance, specifically under diffuse light conditions. Low fabrication cost, semitransparency, flexibility and easiness of assembly are the characteristic features of Dye Sensitized Solar Cells (DSSCs). These specialities distinguish DSSCs from the commercial silicon based photovoltaic devices. So the active research on each component of DSSC to improve its efficiency and lifetime becomes significant^1–4^. Conventional DSSC constitutes four main components photoanode, dye sensitizer, redox electrolyte, and a counter electrode. Here, the wide band gap oxide semiconductor (TiO_2_, ZnO, SnO_2_) based photoanode represents a key component for enhancing the efficiency of DSSCs. There are many reports on the optimization of the nanostructure and selection of photoanode material for the fabrication of DSSCs^1,4–6^. The specific role of photoanode is to transmit the photoelectrons generated by the light absorption of dye sensitizers like Ruthenium based N719 and N_3_ dyes to the external circuit. The redox couple electrolyte restores the original state of the dye by the passage of photoexcited holes. The Pt coated FTO is a widely used promising counter electrode for collecting the photoexcited electrons from the external circuit and for enhancing the catalysis of the electrolyte^1^.

The present work is particularly focused on the fabrication of DSSCs which employ ZnO nanostructures based photoanode exploiting its low cost, easy synthesis, large scale production, as well as good thermal and chemical stability. Furthermore, the higher carrier mobility and diffusion coefficients of ZnO nanostructures ensure a better photovoltaic performance^7–9^. It has been reported that the controlled morphology of photoanode can change the photoconversion efficiency of DSSC^10^. In nanoscience concept of materials, the engineering aspects like dimensional control and design of oriented nanostructures by varying the synthetic strategies are very important^11–14^. All the physical and chemical properties of these nanostructures are altered by the size, shape and surface structure^14,15^. Thus the architecture of nanostructure plays an influential role in the functionality of the material. According to the experimental conclusions, reaction conditions, choice of reactants and medium of reaction can be engineered to diversify the nanostructures^16^. Also, a variety of synthetic approaches including sol–gel synthesis, hydrothermal, co-precipitation, microwave-assisted and laser ablation are proposed for the controlled and synthetic preparation of nanostructures^1,14,15^. Still, there are many other dependent factors which are unclear in connection with the precise and controlled growth mechanism of nanostructures.

Among the transition metal oxide, zinc oxide (ZnO) has an appealing great interest due to its potential applications in various optoelectronic fields. Moreover, it exhibits properties like nontoxicity, thermal and chemical stability, wide band gap energy as well as large exciton binding energy (60 meV)^15,17^. ZnO possess a wide variety of nanostructures including zero, one, two and three-dimensional structures^14,15,17,18^. Many synthetic approaches have been tested by researchers for the preparation of oriented structures. Most of the synthetic approaches have limitations like tedious experimental procedures, high-temperature requirement, and time-consuming processes. Since the controllability and reproducibility of the results are not delivered by most of the experimental techniques, researchers are keen to develop simple and reliable experimental procedures for the synthesis of ZnO nanostructures. Herein, a simple, low growth temperature, ultrasonication assisted solution method is set forward as a better method for the fabrication of self organized ZnO nanostructures. In the solution method, solvents are considered as an enchanting part and the interface-solvent interactions control the aggregation kinetics in the growth. Physicochemical properties of the solvents such as viscosity, dipole moment, polarity, dielectric constant, solubility and coordination ability are expected to influence the structural properties of nanomaterials^19–23^. Lui et al.^24^ studied the solvent effect on the spontaneous growth behavior of uncapped Te nanoparticles and suggested a growth mechanism based on the solvent properties. Several studies have been reported based on the solvent assisted morphological tuning of ZnO nanostructures^21,23,25^. Chen et al.^26^ investigated the influence of polar protic solvents on the morphological tuning of ZnO nanostructures and prepared nanodots, nanodisks, mesocrystals, spheres and sheets using same precursors. They proved that solvent property and structure played an important role in the morphological engineering. In 2018, Liang^27^ successfully tailored the growth of ZnO meso/nanocrystals through a facile precursor hydrolysis process using water/organic solvent (ethanol and DMF) system as reaction media. In the synthesis process, the volume ratio between organic solvents and water plays an important role in developing diverse shapes enclosed by tunable facets. Recently, Arie Wibowo^28^ reviewed the progress of ZnO nanostructures in emerging solar cell applications including dye sensitized solar cells, quantum dot sensitized solar cells, organic and inorganic solar cells and hybrid solar cells. The excellent stability, nontoxicity, proper morphological controllability, low production cost, good reproducibility, and high electron mobility of ZnO will boost their potential application in the emerging photovoltaic market. In one of our previous works^29^, ZnO nanoparticles morphology was successfully controlled using different alcohols as reaction media by an ultrasonication assisted simple solution method without the use of any surfactants. In that report, the physicochemical properties and alkyl group length of the solvents enabled a nanodot to nanorod morphological tuning. Also, we demonstrated the potential use of synthesized structure in the fabrication of DSSCs. Motivated by this work, well defined ZnO nanostructures were synthesized using four solvents such as ethylene glycol, 1-butanol, acetic acid, and water. An investigation on the subsequent aging process of the nanostructures revealed an interesting solvent effect on the growth kinetics. The physicochemical properties of the solvents are given in Table 1. Solvents control the aggregation rate and particle–particle interaction in the reaction kinetics. Solvent properties like dipole strength, coordination capacity, dielectric constant, etc. play important roles in controlling the aggregation rate^13,30^. Here we present a simple synthetic strategy for the preparation of ZnO nanostructures that can be used as the photoanode material in the fabrication of DSSC. The nanostructure dependant enhancement in the internal power conversion efficiency is discussed based on the visible light harvesting capacity and the electron–hole recombination rate.

**Table 1:** Fundamental physicochemical properties of used solvents.

Solvents	Formula	Relative polarity	Dielectric constant
Ethylene glycol	C_2_H_6_O_2_	0.790	38
1-Butanol	C_4_H_10_O	0.586	17.5
Acetic acid	CH_3_COOH	0.648	6.1
Water	H_2_O	1	80

## Result and discussion

**Figure 1:**
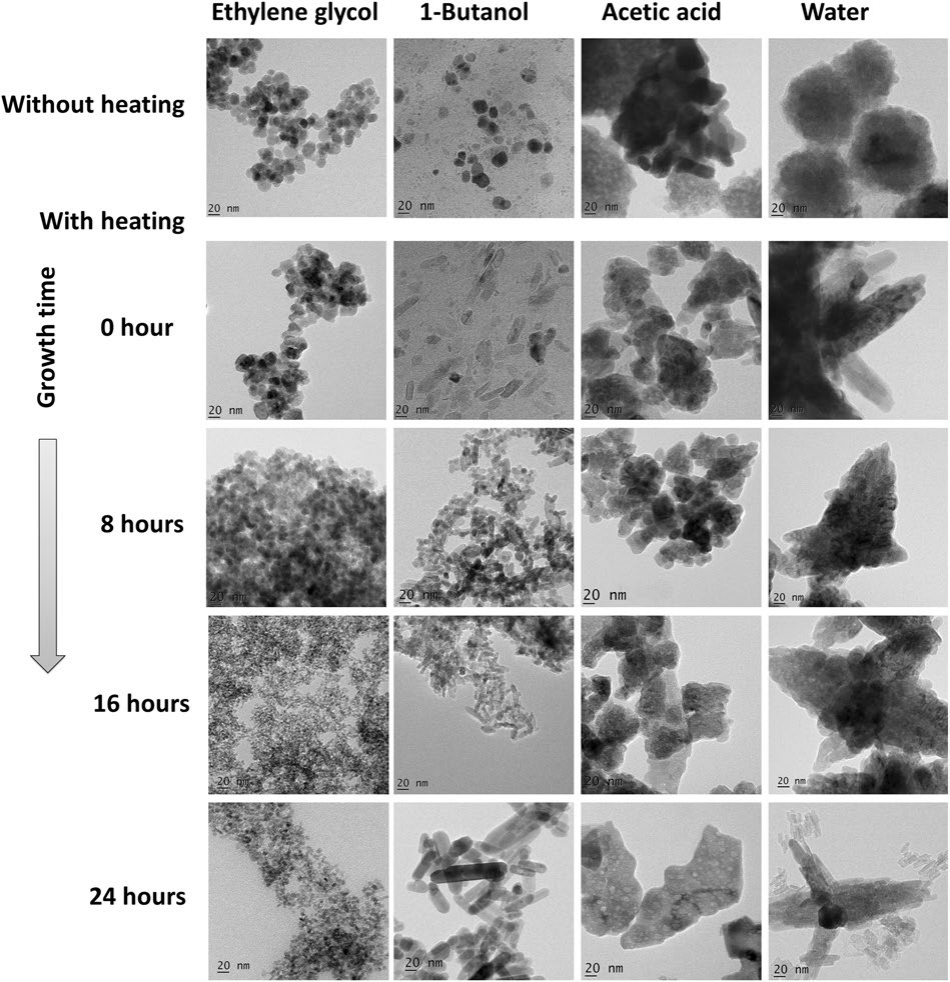
TEM image of ZnO nanostructures synthesized in (**a**) ethylene glycol (**b**) 1-butanol (**c**) acetic acid and (**d**) water.

In order to investigate the formation of ZnO nanostructures, HRTEM and FESEM analysis were carried out. Figure 1 represents the HRTEM images of synthesized ZnO nanostructures and their structural evolution in various solvents. As evident from the figure, the usage of various solvents (C_2_H_6_O_2,_ C_4_H_10_O, CH_3_COOH, and H_2_O) under similar experimental conditions led to the formation of distinct ZnO nanostructures. The figure also depicts the time evolution of ZnO crystalline structures using TEM images for a period of 24 h.

Examination of the morphological aspects of ZnO in C_2_H_6_O_2_ gives an idea about the native growth constraints. It has been observed from Fig. S1 that in the solvent C_2_H_6_O_2,_ ZnO nanostructures maintain spherical morphology before and after heating thereby making the structural evolution independent of the aging time. The morphological properties were identical in the entire series and only a size tuning has been observed (Fig. S2). In the initial stage, well-dispersed spherical nanoparticles with an average size of 17.2 ± 3.5 nm were formed without heating. After heating, (80 $^\circ{\rm C}$, 2 h) a slight increase in the (21.2 ± 4.6 nm) spherical nanoparticle size was noticed due to nucleation. Further, a reduction in ZnO nanodot size was observed with aging time. Particle sizes for the resulting ZnO nanodots were 10.80 ± 5.1 nm, 5.1 ± 0.96 nm and 5.68 ± 0.61 nm corresponding to aging periods of 8 h, 16 h and 24 h, respectively. Unlike the Oswald ripening, we could observe a reduction in the homogenous size distribution of the nanodots with increase in growth time. In the present situation, particle size decreases with an increase in aging, indicating the growth inhibition behavior of the solvent. To verify the solvent inhibitory effect,the morphological aspects of ZnO were examined by varying pH, surfactants, heating time, and temperature of the reaction, which are the major parameters that affect the final product morphology, particle size, number of growth units and nucleation of materials. The evolution of the particle diameter as a function of change in these parameters are shown in the Supplementary Figs. S3, S4, S5 and S6. It can be noticed that the particle shapes are not affected drastically by the variation of parameters and only the size change was observed. In an aqueous solution, homogenous growth is pronounced than heterogeneous growth, indicating the dominant growth controlling nature of the solvent. HRTEM of the nanodots in C_2_H_6_O_2_ prepared by varying the parameters is shown in Fig. S7. The figure indicate that all the particles exhibit a preferential growth along [100] direction of ZnO. The ZnO crystal growth in the plane (100) is less compared to other planes and for ZnO wurtzite structure, (100) nonpolar surface has the lowest surface energy and better stablility^8,14,16,31,32^. Upon variation of all these growth parameters, the ZnO nanodots were found to prefer the growth in a significantly stable (100) surface. Solvent acts as the capping agent to stabilize the growth surface. The shape of the nanoparticles was not changed by the heating and aging process. So the result indicates that C_2_H_6_O_2_ prevents the surface aggregation of ZnO in the growth process and it is a good solvent for synthesizing homogenous ZnO nanodots.

**Figure 2:**
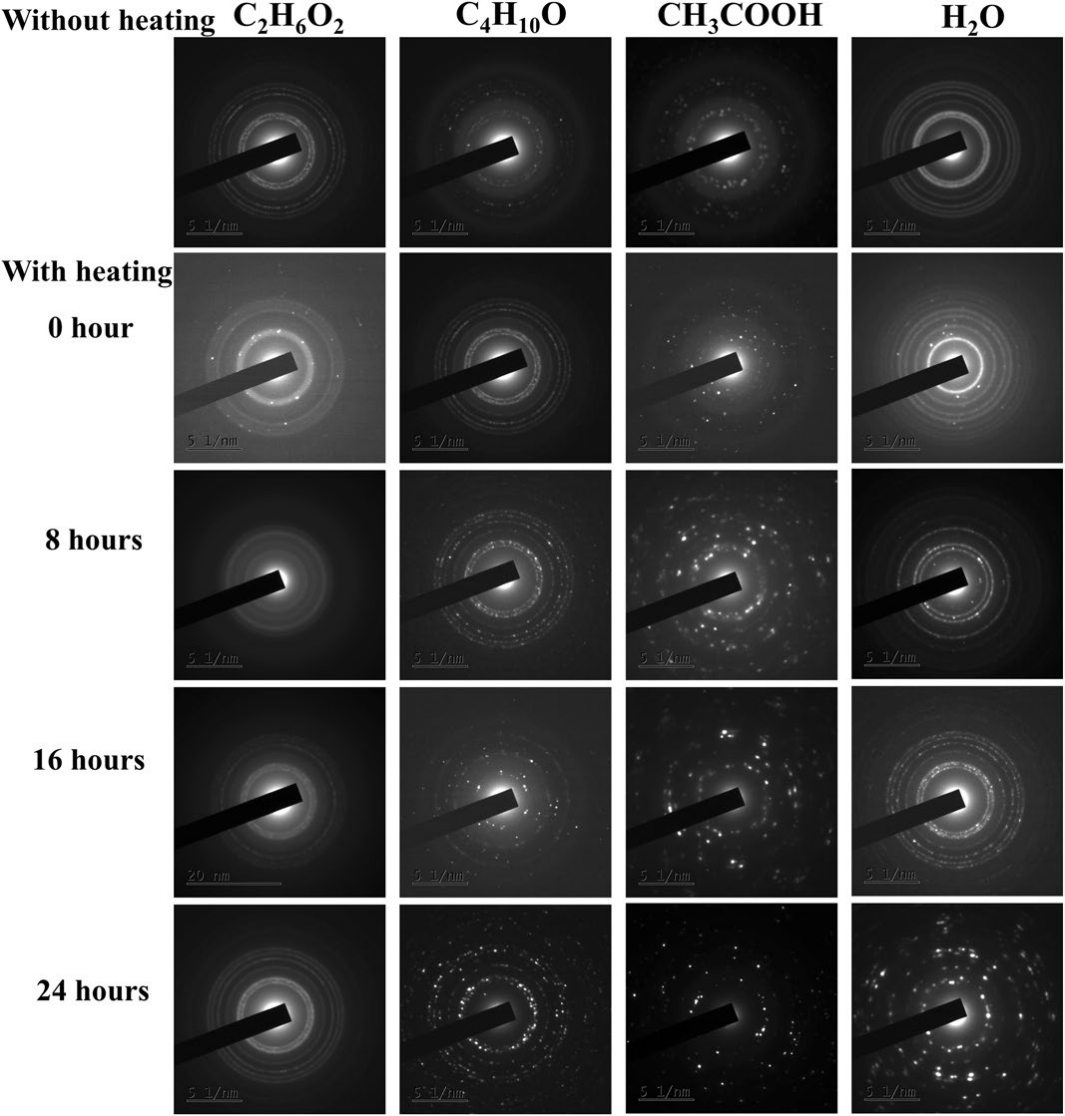
SAED pattern of synthesized ZnO nanostructures in distinct solvents.

**Figure 3:**
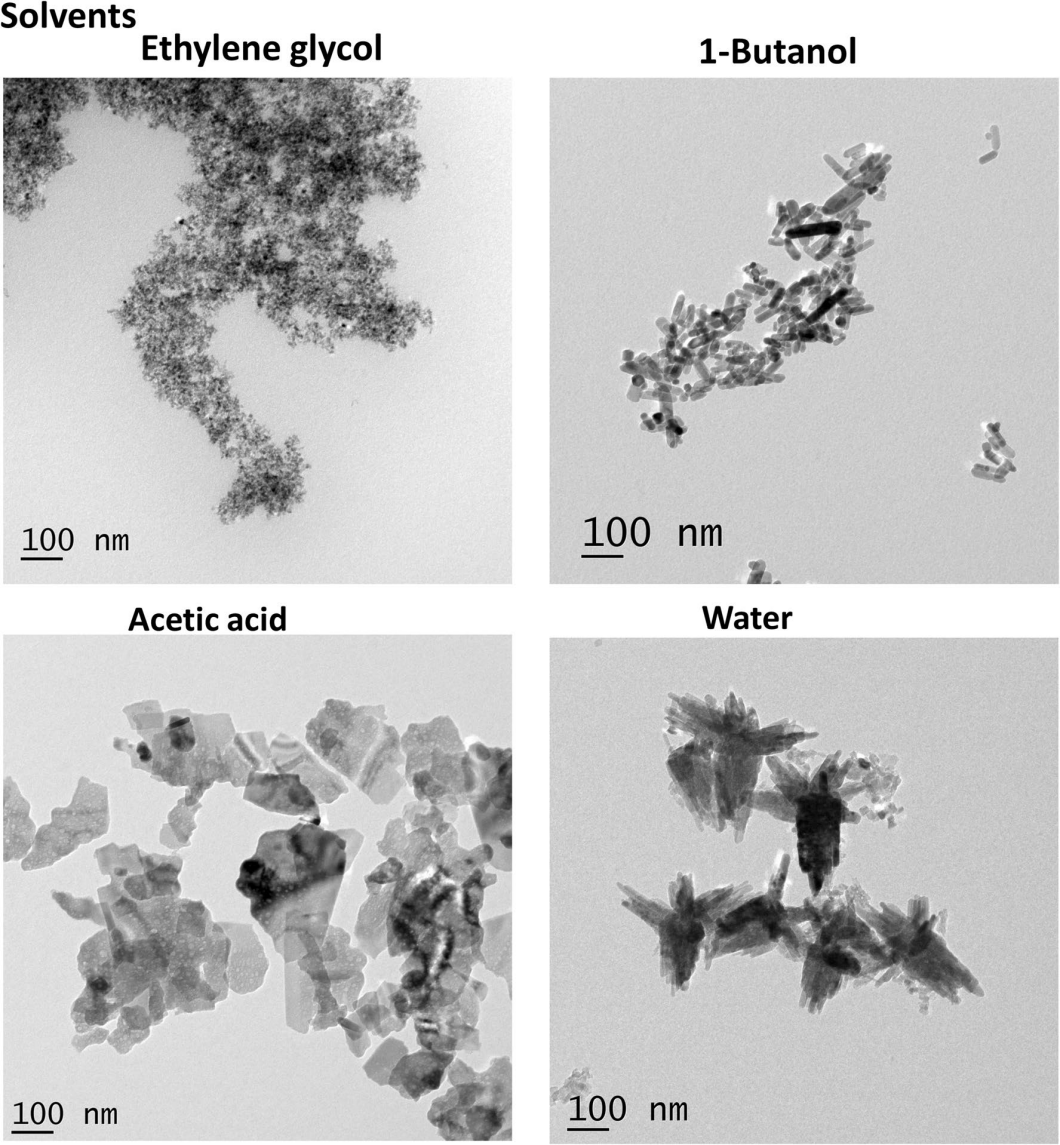
TEM image of synthesized ZnO nanostructures in distinct solvents after 24 h of aging.

Further, we examined the growth behavior of ZnO with C_4_H_10_O as a solvent. When the ZnO sol was heated, the aggregation of nanoparticles allowed the random growth of crystallites under oriented attachment, causing the formation of nanorods^13^. Figure S8 indicates that heat treatment is not enough to form a well-defined structure. The heat treated sol kept for a period of 24 h resulted in crystalline ZnO nanorods indicating that the aging process strongly affects the crystallinity. A similar growth mechanism was also found to occur for the other two solvents, CH_3_COOH and H_2_O as shown in Supplementary Figs. S9 and S10. In both the cases, ZnO aggregates were formed in the initial stage. The heating and subsequent aging process changed the morphology of aggregates giving rise to an ordered structural formation. It was also noticed that in both cases, 24 h aging time is good enough to produce the crystalline structures shown in Fig. 2. ZnO nanoplates were formed in CH_3_COOH and nanoflowers were formed when H_2_O was used as the solvent. Figure 1 shows the excellent control over the shape and size of ZnO nanostructures through aging and by the use of different solvents. For all the solvents under study, 1-day aging resulted in a defined nanostructure and these samples were used for further characterization. Figure 3 shows the TEM image of ZnO nanostructures in various solvents after 24 h of aging. The above findings put forward a cost-effective and simple synthesis method for the preparation of non-identical ZnO nanostructures.

**Figure 4:**
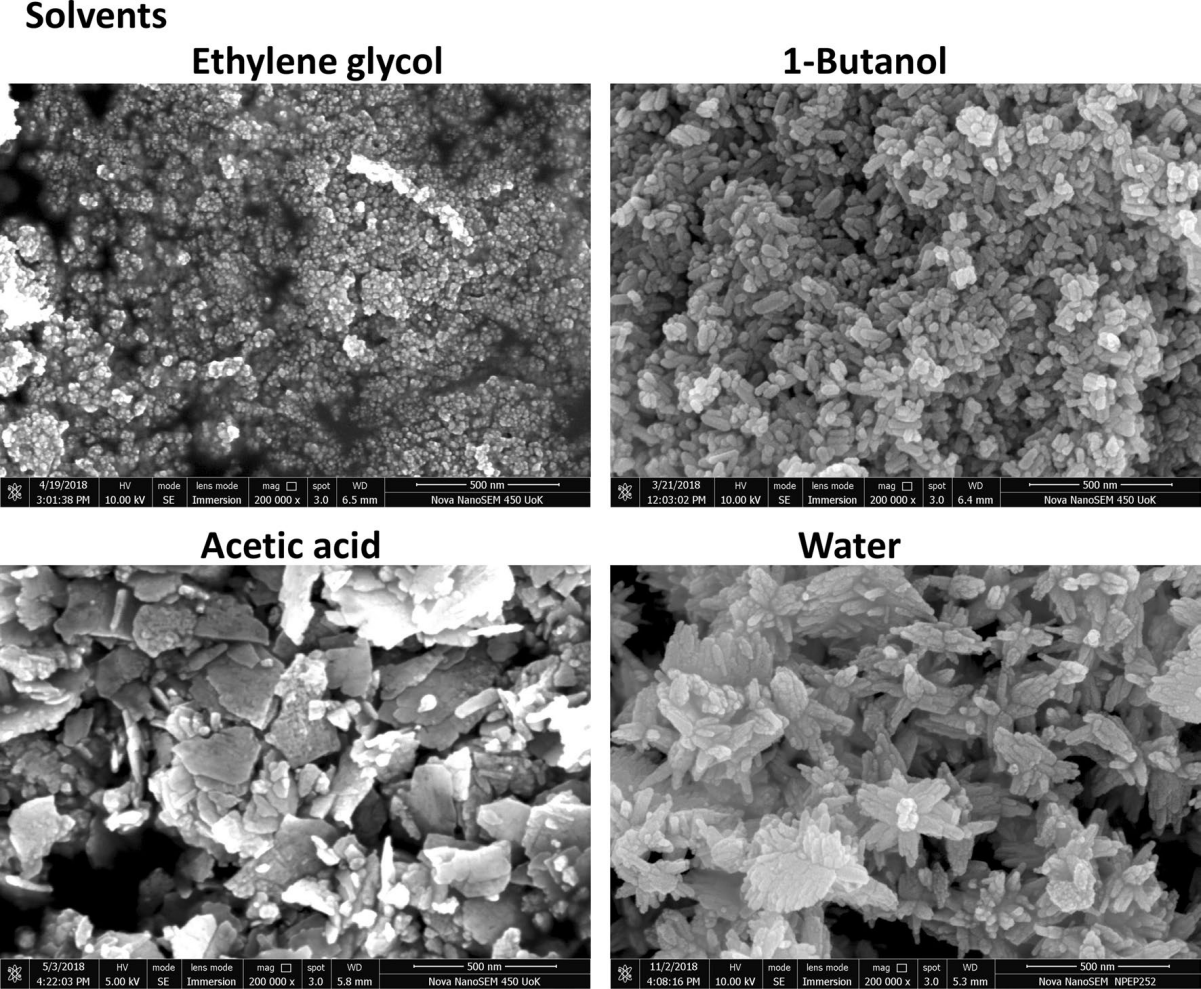
FESEM image of synthesized ZnO nanostructures in distinct solvents after aging for 24 h.

The morphology of the synthesized ZnO nanostructures was further revealed by the FESEM image shown in Fig. 4. In good agreement with the TEM analysis, it reveals the morphological transition from zero dimensional to three dimensional ZnO nanostructures due to the property of the solvents. To investigate the growth orientation and crystalline structures of ZnO, HRTEM and SAED were carried out for each sample. Figure S11 represents the HRTEM image of synthesized ZnO nanostructures. The ZnO nanodots have a lattice spacing of ~ 0.30 nm corresponding to the distance between the (100) planes of ZnO crystal lattice. Other ZnO nanostructures such as nanorod, nanoplate and nanoflower, disclose a d spacing of the lattice ~ 0.25 nm which corresponds to the (101) plane. So all the structures except nanodots exhibit preferential growth in [101] direction because of its fastest growth compared to [100] direction. Selected Area Electron Diffraction (SAED) pattern shown in the Supplementary data Fig. S12, indicates that all the prepared ZnO nanostructures have hexagonal wurtzite nanocrystallite structure with the space group P6_3_mc^33^. The XRD data (Fig. S13) are well-matched with SAED results. All the diffracted, indexed peak positions correspond to the plane (100), (002) and (101) of hexagonal wurtzite structure (JCPDS No: 01-089-1397). The XRD patterns of all the structures show that neither there is any change in the peak positions nor there any impurity peak.

### Growth kinetics of ZnO in various solvents

**Figure 5:**
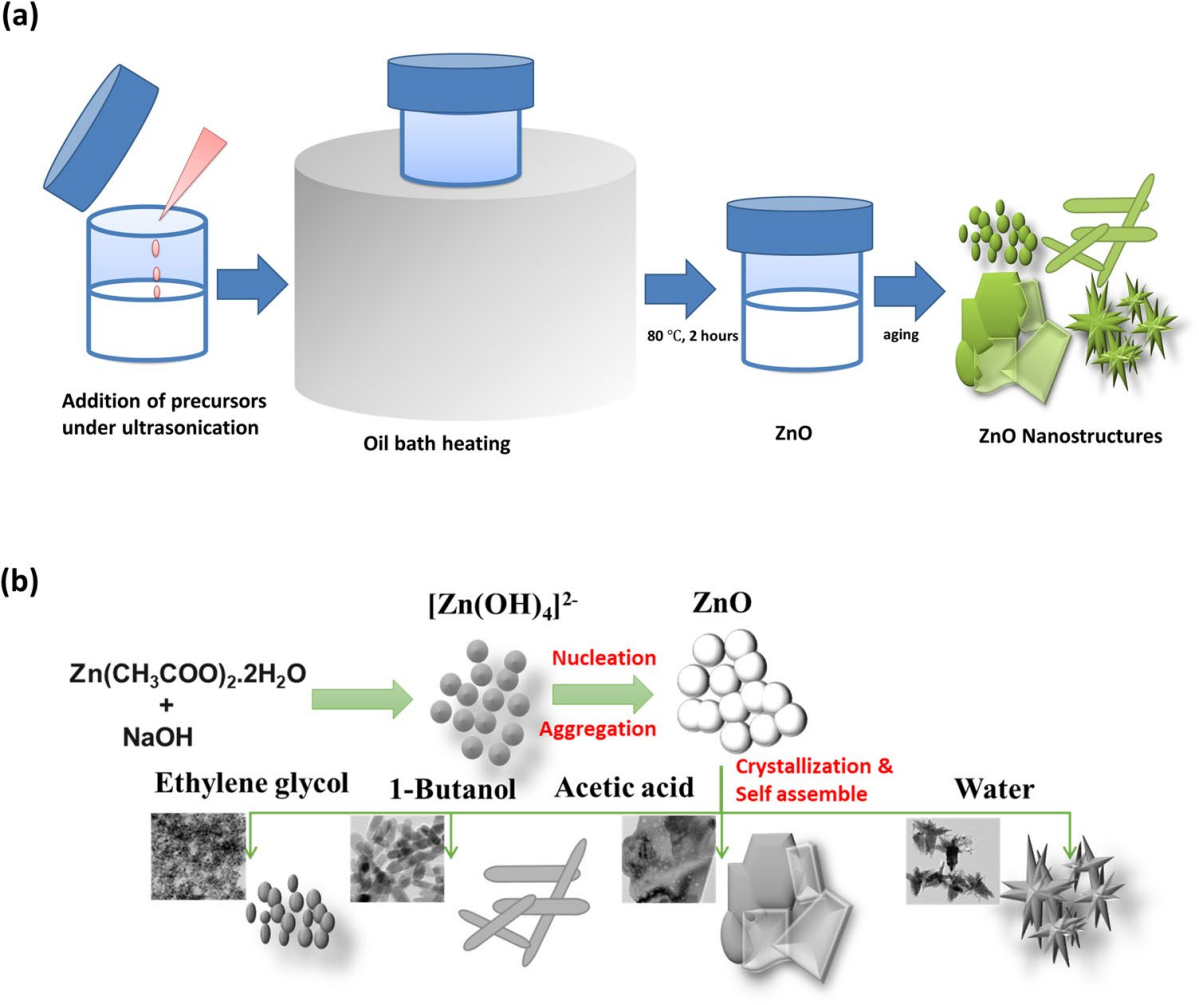
Schematic diagram of (**a**) synthesis procedure (**b**) growth mechanism of ZnO nanostructures in the distinct solvents.

Crystal growth mechanism in various solvents underwent distinct growth behavior and the notable differences in the growth kinetics are shown in the schematic diagram Fig. 5. Interestingly, the tuning of the ZnO nanostructure morphology was achieved by using four distinct reaction media. Evidently in the selected solvents, a well-defined nanostructure of ZnO evolved under 24 h of aging. Since the solvent properties significantly affect the growth kinetics and aggregation, a detailed description of the interaction is needed to understand the distinct growth mechanism. The crystal growth in solution mainly includes two stages (1) nucleation process (2) growth process^5,16,34–37^. In the nucleation stage, OH^−^ ions react with Zn^2+^ ions in solvents to form Zn(OH)_4_^2−^ growth unit. ZnO nuclei of a few nanometer sizes are formed by the decomposition of the growth unit. The secondary growth process is more complicated than the primary nucleation process in which primary ZnO nanoparticles will be assembled to form various morphologies. Solvent polarity, dielectric constant, supersaturation of ZnO primary particles in the solvent and solvation degree of NaOH are the major factors that affect the growth mechanism. Other than these parameters, the electrostatic force plays a critical role in the growth of ZnO nuclei^29,38^.

The repulsive electrostatic interaction energy is,

$$E= \frac{1}{4\pi \varepsilon }\frac{{Q}_{1}{Q}_{2}}{r}$$

where ε is the dielectric constant of the medium, Q_1_ and Q_2_ are the charge value of the two particles, r is the separation between two particles. The coloumbic force being repulsive, hinders the aggregation of nanoparticles leading to a decrease in the growth rate. This interaction energy is directly proportional to the charge value of ZnO nanoparticles and inversely proportional to the dielectric constant of the solvent molecules. In the present experiment, no surfactants were added to the solvents. The charge value of ZnO nanoparticles is derived from the ionization of the solvent molecules, which is closely related to its polarity. So, the distinct ZnO growth behaviour depends directly on the polarity and inversely on the dielectric constant of solvent molecules. The literature suggests that a faster growth rate of the nanostructure is observed in weakly polar solvents^29,39^.

By comparison, it was found that the growth is enhanced according to the dielectric constant in the case of H_2_O and C_4_H_10_O. Both the solvents possess a high dielectric constant (ε) value. Compared to other solvents selected, polarity value is lesser in C_4_H_10_O and one-dimensional nanorod structure was formed. In the case of C_2_H_6_O_2_, a growth inhibition nature was observed. Compared to C_4_H_10_O and CH_3_COOH, C_2_H_6_O_2_ possess higher polarity leading the inhibitive nature and the formation of nanodot. Among the four selected solvents, H_2_O possess the highest polarity resulting in increased repulsive interaction between solvent and ZnO nanoparticles. At the same time, the dielectric constant of water is sufficient to reduce the repulsive interaction. Thus in water, there is enhanced ZnO growth in the direction [101], forming a nanoflower structure. The acetic acid with smaller dielectric constant and polarity exhibit a faster growth. In order to explain this contradictory process, we measured the zeta potential of the colloidal solution.

According to the literature,the zeta potential is also a key factor that controls the growth mechanism. It also has the nature of a repulsive force that can hinder the growth rate^30^. The measured value of zeta potential for the ZnO nanostructures are found to be − 26 mV, − 7.2 mV, − 4.5 mV and − 2.5 mV for C_2_H_6_O_2_, C_4_H_10_O, CH_3_COOH and H_2_O, respectively as shown in Supplementary Fig. S14. The general mechanism is an electric double layer around the colloidal ZnO nanoparticles inhibiting the aggregation/agglomeration rate. As a result, the highest absolute value of zeta potential causes a strong hindering effect in the growth. According to the measured zeta potential values, the particle movement is strongly hindered in C_2_H_6_O_2_ and enhanced in H_2_O. This inference is in agreement with the observations from the morphological characterization techniques such as TEM and FESEM. For CH_3_COOH, the zeta potential value is found to be lesser compared to that of C_4_H_10_O. Hence in this case, the growth kinetics favours the formation of 2 dimmensional plate like structures. So, polarity, dielectric constant and zeta potential of the colloidal solution are the three parameters that can completely explain the interactions and growth kinetics of the ZnO nanostructures. The measured values of these parameters are depicted in Supplementary Table S1.

**Figure 6:**
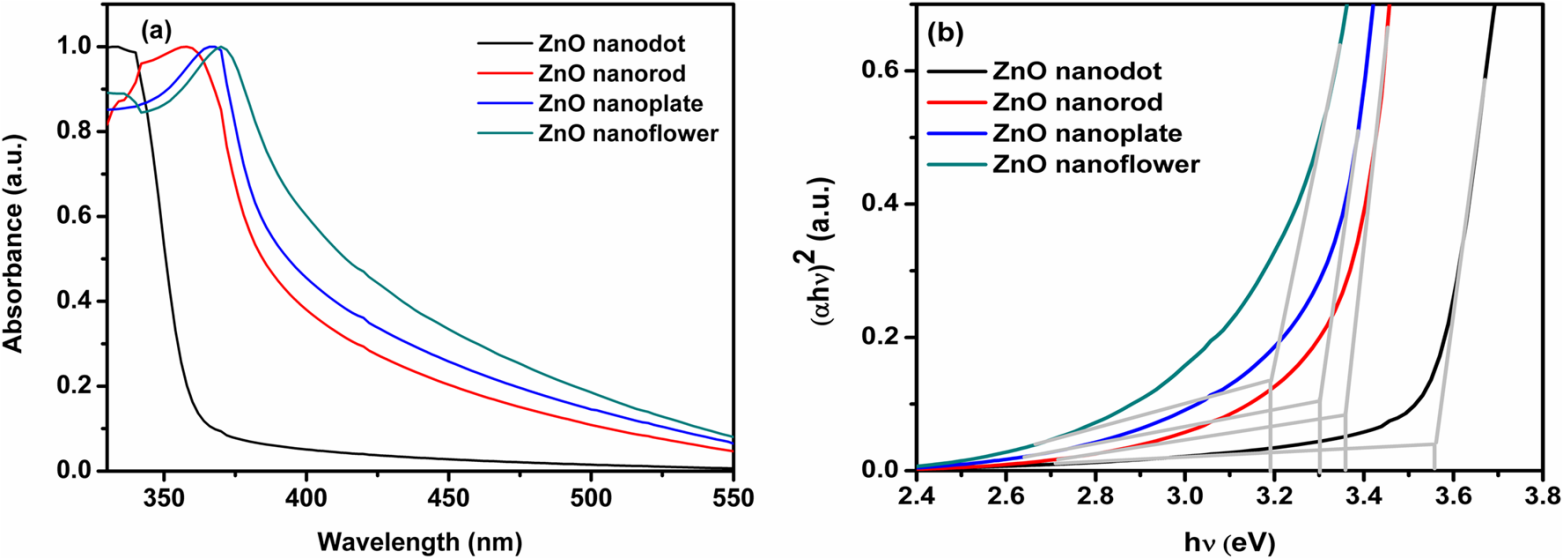
(**a**) UV absorption spectra (**b**) estimation of bandgap of ZnO nanostructures synthesized in distinct solvents.

**Figure 7:**
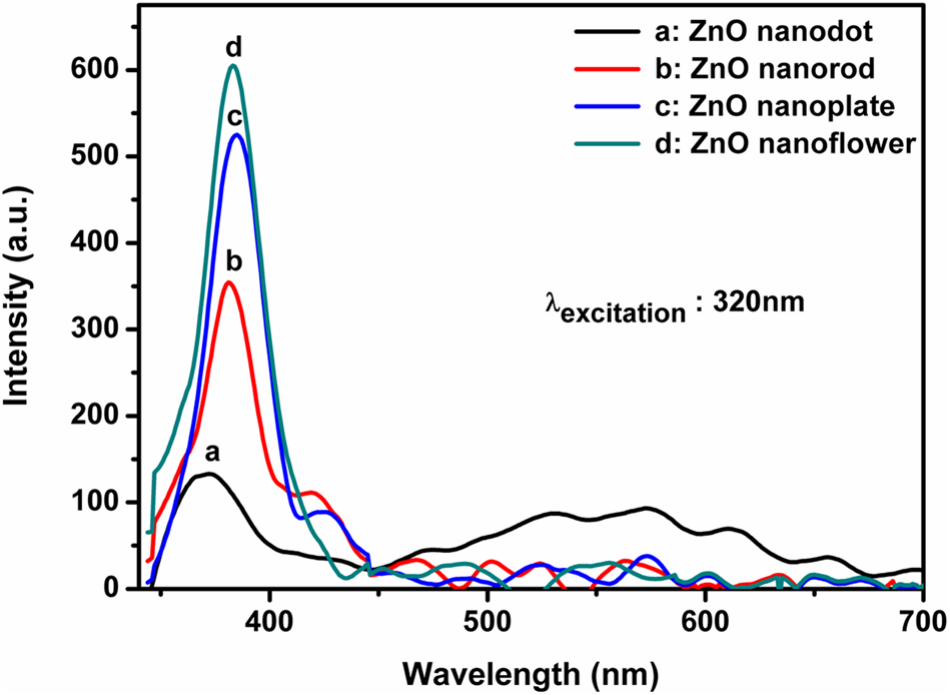
PL emission spectra of ZnO nanostructures synthesized in distinct solvents.

The optical properties of ZnO nanostructures were studied by UV–Vis spectroscopy and the results are presented in Fig. 6a. It is clear that ZnO nanodots show a significant blue shift of optical absorption edge due to the quantum confinement effect. The optical bandgap diagram shown in Fig. 6b was used to evaluate the optical bandgap utilizing the Taue plot relation ${(\alpha h\nu )}^{2}=A(h\nu -{E}_{g})$, where α is the absorption coefficient, $h\nu$ is the photon energy, ${E}_{g}$ is the bandgap energy and A is a constant^40,41^. The estimated values of bandgap were found to vary from 3.2 to 3.5 eV for particle dimensions zero to three. The UV absorption peak of the nanodot shows a blue shift indicating an increase in the bandgap. The estimated hydrodynamic radii of the synthesized ZnO nanostructures are shown in Fig. S15. The emission properties of the samples were analyzed using photoluminescence spectra recorded at an excitation wavelength of 320 nm as shown in Fig. 7.

Generally, ZnO near band edge emission spectra arises due to free exciton recombination and excitons related to intrinsic defects. Most commonly seen defect states are V_O_ (Oxygen vacancies), V_Zn_ (Zinc vacancies), Zn_i_ (Zinc interstitials), O_i_ (Oxygen interstitials), O_Zn_ (Zinc antisites) and Zn_O_ (Oxygen antisites)^15,42–45^. PL studies of ZnO nanostructures at room temperature give rise to an intensity varied narrow UV-emission peak. In addition to the UV emission, an equally intense broad range visible emission was also found to occur for ZnO nanodot. The broad visible emission is attributed to the presence of defects states. There are numerous reports investigating the role of different defect states on visible emission. According to these studies, the deep level defects of zinc vacancies, oxygen vacancies are responsible for blue emission, oxygen vacancies for green emission, oxygen interstitials for yellow emission and zinc interstitials for red emission^11,15,45^. The above observations lead to the conclusion that ZnO nanodot possesses more lattice defect states compared to other structures.

To gather more understanding about the defects, Raman spectroscopy was performed on the synthesized ZnO nanostructures at room temperature under visible light excitation. In the case of wurtzite ZnO crystals of group ${C}_{6v}^{4}$, the optical phonons at the Brillouin Zone Γ are represented as

$$ \Gamma = {1}A_{{1}} + { 2}B_{{1}} + E_{{1}} + {2}E_{{2}} $$

where, Raman active optical modes are ${A}_{1}$, ${E}_{1}$ and ${E}_{2}$ modes; ${B}_{1}$ is a silent mode. The ${A}_{1}$ and ${E}_{1}$ are the two polar branches that consist of longitudinal and transverse optical components. The nonpolar ${E}_{2}$ branches are split in to high and low-frequency modes^15^. Figure S16 shows the Raman spectra of ZnO nanostructures. In this configuration, the ${E}_{2}$ high and low modes are found to arise dominant for all structures. As indicated by the XRD results, these are modes with high crystallinity of the wurtzite structure of ZnO. Also, we noticed that a peak centered at 583 cm^−1^ is found only for ZnO nanodots. This peak corresponds to ${E}_{1}\left(LO\right)$ modes originated from the second-order Raman scattering. This mode has been associated with the presence of excess Zn interstitials indicating the presence of structural disorders^8,15,18,46^.

**Figure 8:**
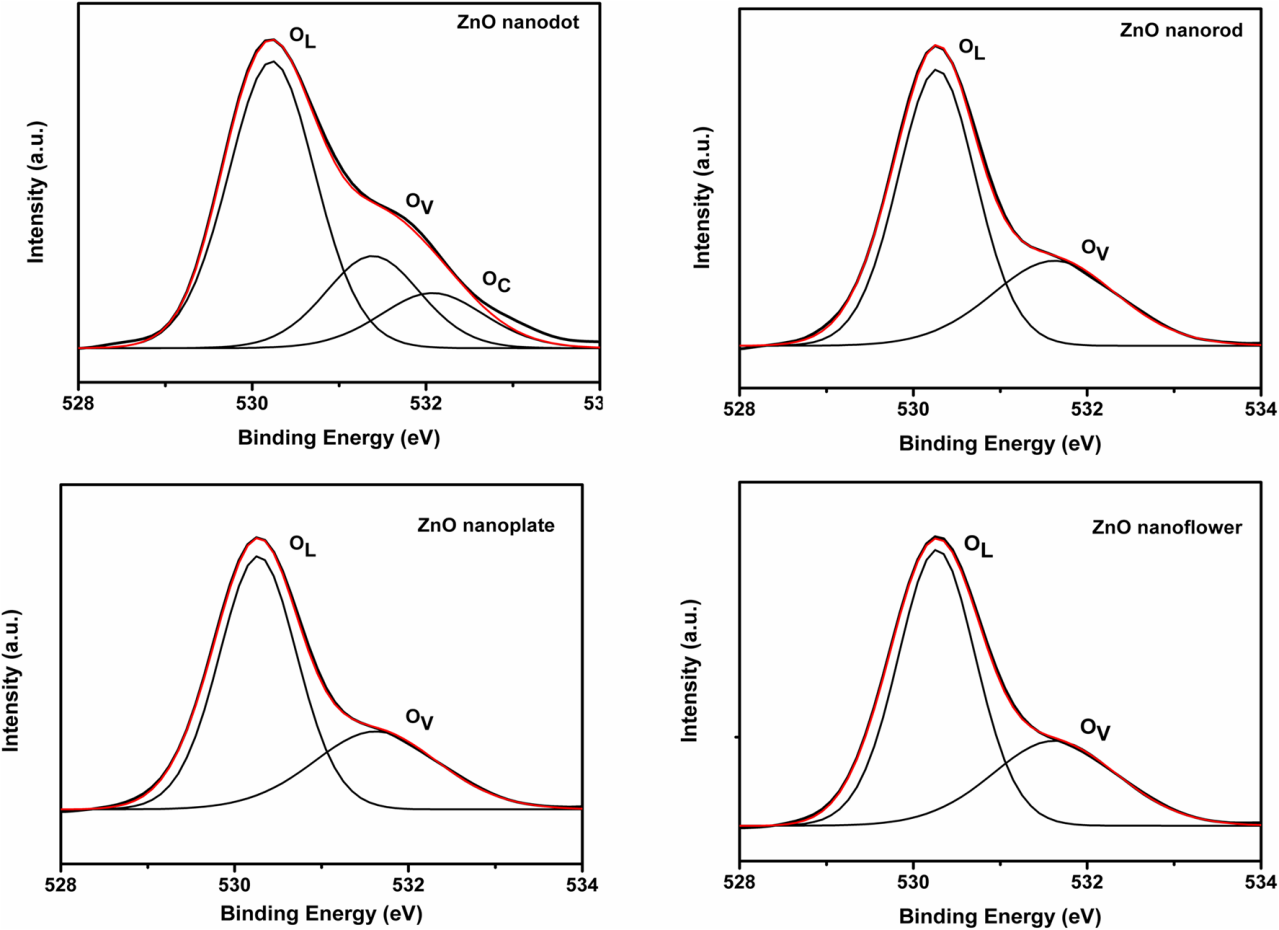
XPS spectra of ZnO nanostructures synthesized in distinct solvents.

To gain a better understanding of the surface defect states of ZnO nanostructures, XPS was performed. Figure 8 shows the deconvoluted O1s spectra. For all the samples, the peak at 530 eV is highly intense and assigned to O^2−^ ions in the Zn–O bonds of wurtzite ZnO crystal structure (O_L_). The second peak at 531 eV is due to the defect state (O_V_). The oxygen spectra can be fitted to the three peaks at ~ 530 eV, ~ 531 eV, and ~ 531.7 eV for the ZnO nanodots. The peak at 531.7 eV can be assigned to the surface adsorbed oxygen species (O_C_). This third peak indicates the presence of more defect states in the dots like structure^29,47^. The result agrees well with PL and Raman interpretations. Thus zero to three dimensional nanostructures of ZnO were successfully prepared. In the next step, synthesized nanostructures were used as photoanode material in the fabrication of DSSCs.

### Cell performance

**Figure 9:**
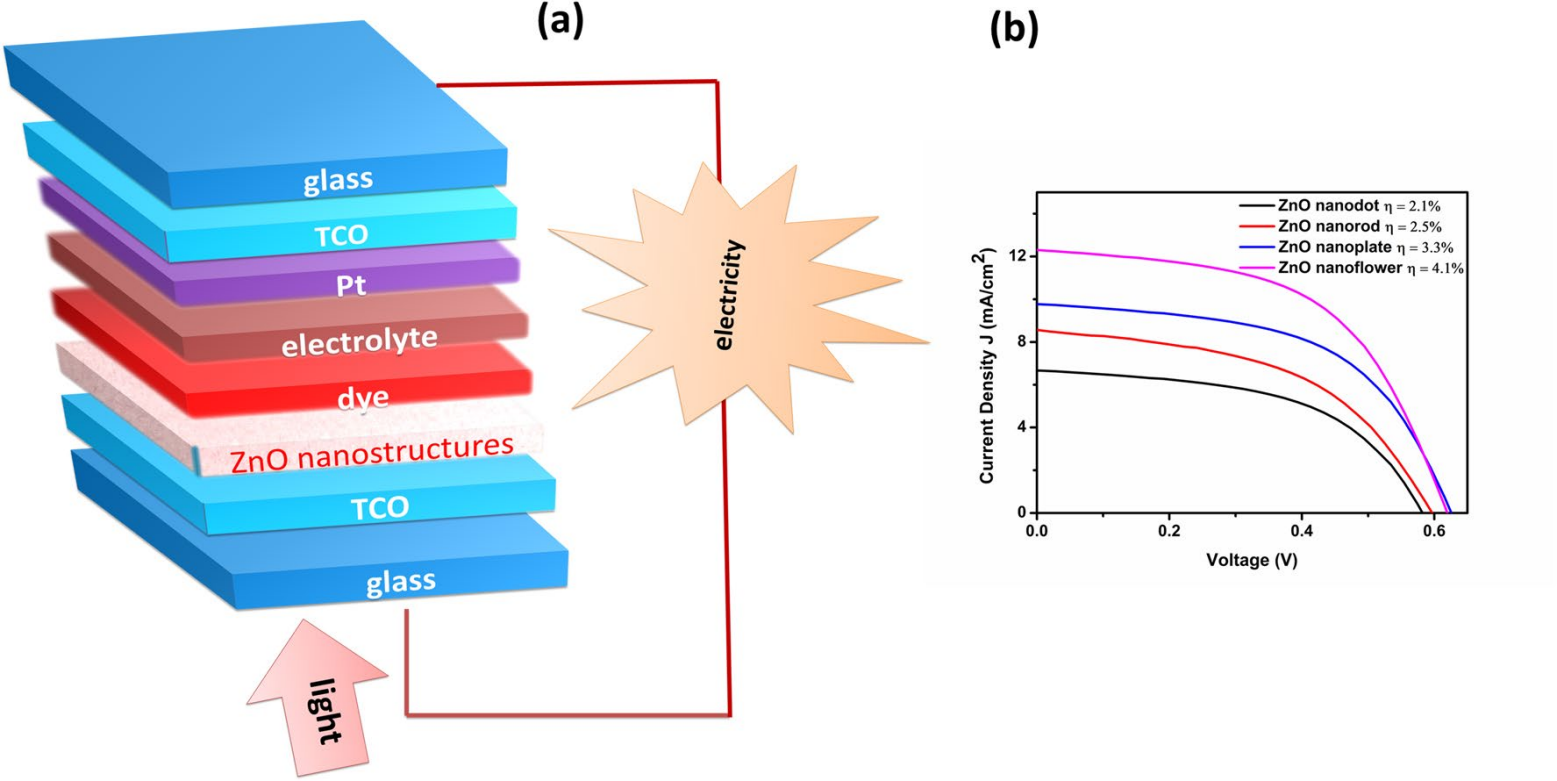
(**a**) Schematic representation of DSSC and (**b**) J–V curves of fabricated DSSCs usingZnO nanostructures as photoanode.

Solar cells consisting of synthesized ZnO nanostructures as photoanode material were fabricated and tested under AM 1.5 illumination. Figure 9a presents the schematic representation of the proposed DSSC structure. The performance characteristics of the cells are presented in Fig. 9b in terms of short circuit current density (J_SC_) and open-circuit voltage (V_OC_). It is found that all the fabricated cells exhibited approximately similar V_OC_ value. However, it can be noted that the J_SC_ value largely depends on the morphology of the nanostructures. The measured J_SC_ for the four solar cells making use of nanostructures; dot, rod, plate and flower were found to be 6.68, 8.60, 9.80 and 12.32 mA cm^−2^, respectively. The power conversion efficiency (PCE) of cells was also found to follow a similar trend as that of J_SC_ values. An enhancement in PCE was observed with increment in J_SC_ value in the order nanoflower > nanoplate > nanorod > nanodot. Among all, the cell incorporating the nanoflower was found to exhibit the highest current density as well as efficiency. The efficiency of the cell with ZnO nanoflower based photoanode was found to be 4.1%. It induces strong visible photon capturing by enhancing the incident light scattering and weakening the transmittance. The ordered large-sized nanostructures inevitably cause an enhancement in light scattering and a decrease in the charge-transfer resistance. Thus they exhibit a superior photovoltaic performance as compared with that of small grain sized nanoparticles^48^. Figure S17 shows the reflectance spectra of ZnO nanostructure films wherein the nanoflower film show higher reflectance than the other structures. Generally, higher reflectance indicates a better light scattering ability, which implies a longer light path inside the photoanode thereby offering a better opportunity for the dye molecules to absorb more photons. The random distribution of growth direction of nanoflowers can also significantly improve the light trapping effect^4–6^. Moreover, the surface area plays a vital role in the dye adsorption and light scattering. Table 2 shows the direct correlation between nanostructure surface area and dye adsorption. The increased surface area enhances the dye adsorption on the photoanode surface and faster dye penetration during the sensitization process, giving rise to an increased efficiency^5^. An estimation of the adsorbed amount of dye was carried out using the method reported previously^49^ and the corresponding UV absorbance spectra are shown in Fig. S18. The above-discussed factors such as higher scattering ability, larger surface area, and enhanced dye adsorption improve the efficiency of a cell fabricated using ZnO nanoflower. ZnO nanoplate based DSSC displayed second highest efficiency (3.3%) followed by the device based on ZnO nanorod (2.5%) and nanodot dye sensitizied solar cell (2.1%).

**Table 2:** The photovoltaic parameters of DSSCs fabricated using synthesized ZnO nanostructuresas photoanode.

ZnO nanostructures	V_OC_ (V)	J_SC_ (mA cm^−2^)	FF	ɳ (%)	Dye adsorption (nmol cm^−2^)	BET surface area (m^2^ g^−1^)	${\tau }_{e}$ (ms)
Dot	0.58	6.68	0.50	2.1	22.1	19.26	1.5
Rod	0.59	8.60	0.50	2.5	28.9	27.67	2.19
Plate	0.63	9.80	0.54	3.3	44.6	40.91	2.36
Flower	0.62	12.32	0.54	4.1	50.4	60.39	3

**Figure 10:**
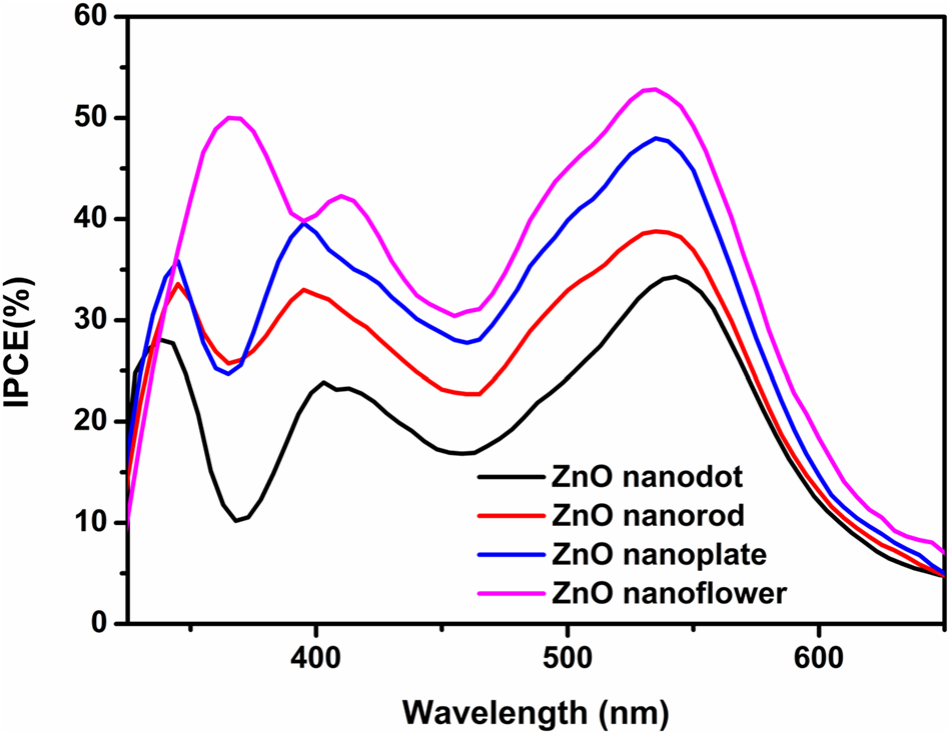
IPCE spectra of DSSCs based on ZnO nanostructures as photoanode.

Figure 10 depicts the dependence of incident photon to current conversion efficiency (IPCE) on the illumination wavelength for the ZnO nanostructure based DSSCs. The IPCE spectra of DSSCs were found to follow a similar trend as the J–V curve described in Fig. 8. In the IPCE spectra, the absorption maximum for the N719 dye occurs in the visible region around 530 nm with values of 34%, 38%, 47% and 52% for nanodot, rod, plate, and flower, respectively. Clearly, IPCE obtained for ZnO nanoflower is almost 1.5, 1.4 and 1.1 times the corresponding values obtained for dot, rod and plate structures, respectively. This result can also be correlated with the higher J_SC_ value and better light scattering effect applicable to the flower structure.

**Figure 11:**
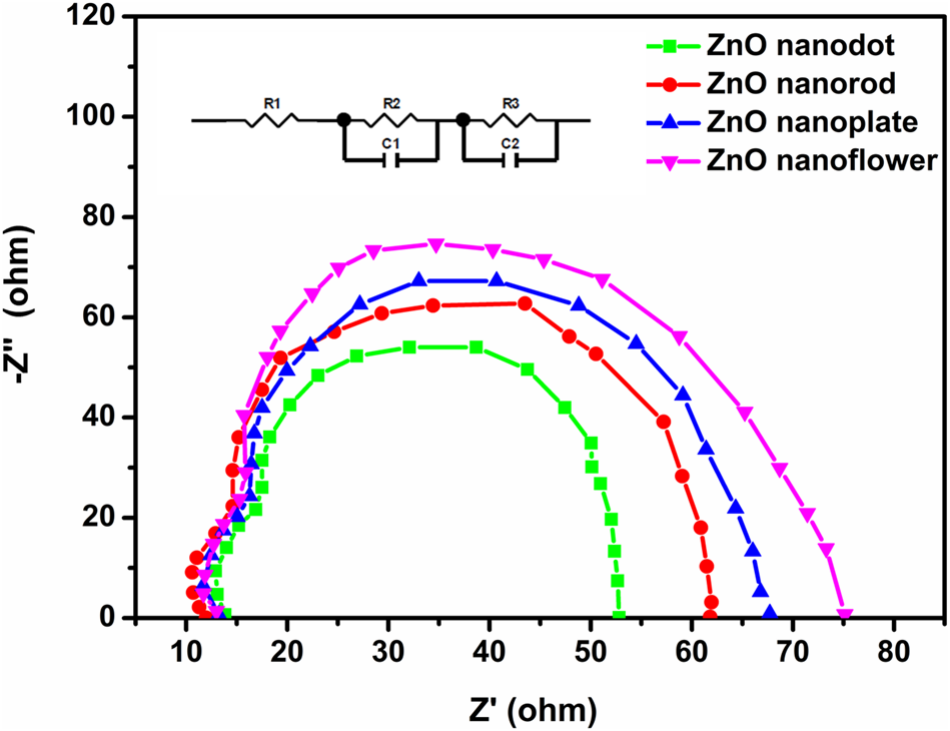
Nyquist plots of fabricated DSSCs with ZnO nanostructures as photoanode.

Electrochemical impedance spectroscopy (EIS) is a powerful experimental technique for gaining better insight into the interfacial charge transfer processes within DSSCs. The EIS was performed under the dark condition at a bias voltage equal to V_OC_. The Nyquist plots for the fabricated DSSCs with various ZnO nanostructures as photoanode material are shown in Fig. 11. Two semicircles are visible in the higher and middle-frequency regions. The small semicircle is assigned to the redox reaction at the platinum counter electrode and larger semicircles take into account the charge transfer mechanism at the photoanode/dye/electrolyte interface^1,2,6,48,50^. The middle arc is larger for the fabricated DSSCs indicating an increase in the recombination resistance and a reduction in the recombination probability at photoanode/dye/electrolyte interface^3^. The charge recombination resistance (R_rec_) between the injected electron at the oxide surface and the electron acceptor ion ${I}_{3}^{-}$ in the electrolyte can be deducted by fitting the middle-frequency semicircle using Z-view software. The value of R_rec_ is directly related to the diameter of the semicircle. An increase in arc diameter increases the recombination resistance between the electron in the photoanode and hole in the electrolyte. In Fig. 11, the apparent increasing trend of the central arc diameter is evident with respect to the dimension of photoanode material in the order nanoflower > nanoplate > nanorod > nanodot. The result clearly indicates the increased interfacial charge recombination resistance of ZnO nanoflower based DSSC in comparison with the device based on other structures.

**Figure 12:**
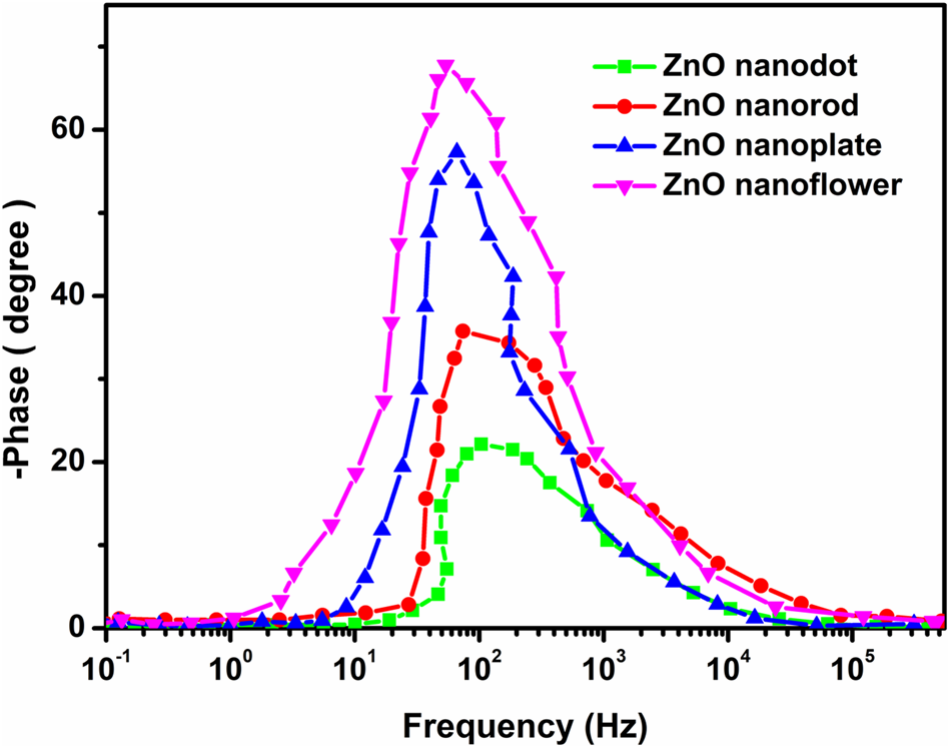
Bode-phase plots of fabricated DSSCs with ZnO nanostructures as photoanode.

The values for R_rec_ for nanodot, nanorod, nanoplate, and nanoflower based DSSCs were estimated to be 81, 93, 106 and 129 Ω, respectively. However, the experimental results indicate that the recombination rate depends on the open-circuit voltage (V_OC_). The significant increase in recombination resistance of the cell, retarding charge recombination between injected e^−^ and I_3_^−^ ions in the electrolyte, offers a higher value of V_OC_ and fill factor (FF)^49^. The measured photovoltaic parameters of the DSSCs for different photoanodes are depicted in Table 2. The gradually increasing recombination resistance also results in longer electron lifetime. The corresponding electron lifetimes (τ_e_) were also estimated from Bode-Phase plots^3^ shown in Fig. 12, using the following equation:

$${\tau }_{e}=\frac{1}{{\omega }_{max}}=\frac{1}{{2\pi f}_{max}}$$

The cell with ZnO nanoflower as the photoanode material exhibits a higher ${\tau }_{e}$ which favors longer electron transport distance with less diffuse hindrance. This results in an effective and significant electron capture leading to a better device performance^48,51^. The above-discussed factors help in enhancing the photovoltaic performance of the ZnO nanoflower structure. The photovoltaic parameters of the fabricated DSSCs are depicted in Table 2.

The performance of the fabricated cells was also compared with standard TiO_2_ based cells the details of which are described in our previous report^29^. For the TiO_2_ photoanode based cell the obtained maximum efficiency was found to be 3.82%. In the present study, the cell fabricated using nanoplate and nanoflower photoanode show nearly similar and higher efficiency than the standard TiO_2_ based cell. Thus the present study suggests that the ZnO nanostructures are good substitutes for TiO_2_ in DSSC fabrication_._The report contributes to the scientific society by proposing a simple and consistent synthesis method for ZnO nanostructures that can offer excellent performance in DSSCs.

In summary, a simple synthesis method is developed for the fabrication of ZnO nanostructures making use of four different solvents and subsequently optimizing them as the effective photoanode material in DSSC architecture. The structural evolution and growth kinetics of ZnO nanostructures in various solvents was also investigated. The findings provide new insights into the influence of solvent physiochemical properties and zeta potential in the growth process. The prepared ZnO nanostructures in zero to three dimensions were successfully incorporated as photoanodes in DSSCs. The results evidently prove the significance of the distinct nanostructures in influencing the photovoltaic parameters. The photovoltaic performance of DSSCs based on nanoflower as well as nanoplate was superior to the devices based on nanodots and nanorods not only in conversion efficiency but also in light-scattering capability and charge-transfer resistance. The results contribute a simple, consistent, cost-effective and feasible solution method for designing appropriate ZnO nanostructures in distinct dimensions for the fabrication of efficient photoanodes.

## Experimental part

### Synthesis of ZnO nanostructures

ZnO nanostructures were synthesized by integrating a simple solution method with ultrasonication. In a typical synthesis of ZnO nanostructures, 0.1 M Zinc acetate dihydrate (Zn(CH_3_OO)_2_·2H_2_O, Sigma Aldrich, 99.999%) solution and 0.5 M Sodium hydroxide (NaOH, Merck) solution were mixed dropwise under ultrasonication (EQUITRON, 53 KHz, 100% power factor). After 10 min of sonication a white transparent precipitate was formed which was heated in an oil bath at a constant rate to a temperature of 80 $^\circ{\rm C}$ for 2 h. The dispersion media used for the above synthesis were ethylene glycol (C_2_H_6_O_2_), 1-butanol (C_4_H_10_O), acetic acid (CH_3_COOH) and water (H_2_O).

### Investigation of morphological evolution

For the investigation of the structural evolution of uncapped ZnO nanoparticles, the colloidal solution of ZnO obtained after heating was kept in a dark chamber. At equal intervals of times, the morphological evolution of ZnO nanoparticles was investigated using TEM analysis. The effect of heating on the structural evolution of ZnO nanoparticles was also studied by comparing the TEM images of white transparent precipitate before and after heating.

### Characterization of structure and morphology

XRD studies of the as synthesized ZnO powders were carried out using a Bruker AXS D8 Advance X-ray diffractometer. The morphology of the resultant products were analysed using high resolution transmission electron microscopy (HR-TEM Model: JEM 2100) and Nova Nano Field Emission Scanning Electron Microscopy (FESEM). The particle size and zeta potential of ZnO nanocolloids were calculated using HORIBA SZ-100 particle size analyser. The elemental and chemical states of the samples were evaluated by X-ray Photoelectron Spectroscopy (XPS KRATOS Axis ultra system). A Brunauer–Emmett–Teller (BET Tristar-3020) was used for the characterization of surface area of the resultant products. JASCO V-570 UV–Vis NIR spectrometer and Cary Eclipse Varian Spectrofluorometer were used for recording absorption and emission spectra respectively.

### Photovoltaic characterization

The as prepared sample of ZnO nanostructures was used for the photoanode fabrication. In the sandwich type DSSC fabricated, a Pt coated FTO glass act as a counter electrode. 0.5 mM N719 dye (Solaronix SA, Switzerland) and Iodolyte electrolyte were also used in the fabrication process. The detailed description of the fabrication process is discussed in our previous report^29^. The unique paste of ZnO nanostructures were coated on FTO using the doctor blade technique and was sintered at 450 $^\circ{\rm C}$ for 3 h. The resulting photoanodes were soaked in 0.5 mM N719 dye solution for 10 h. After washing with ethanol, the photoanode and Pt-counter electrode were sandwiched together by hot pressing. Iodolyte electrolyte was filled between the two electrodes having an active surface area of 0.25 cm^2^. The Current–Voltage characteristics of the fabricated cell were studied using a Keithley 2400 source meter under simulated AM 1.5G illumination (100 mW cm^−2^) provided by a solar light simulator (Newport Corporation). The IPCE (Internal Power Conversion Efficiency) spectra were measured as a function of wavelength using a PVE 300 photovoltaic QE system. The Electrochemical Impedance Spectra (EIS) were analyzed with a CH electrochemical workstation.

## Supplementary Information

Supplementary Information.
