# Supplementary material for: Solvent assisted evolution and growth mechanism of zero to three dimensional ZnO nanostructures for dye sensitized solar cell applications
Source: Sci Rep. 2021 Mar 17;11:6159. doi: 10.1038/s41598-021-85701-9 (PMC7969771; doi:10.1038/s41598-021-85701-9)
Supplement: Supplementary file 1 — Supplementary Information. [file 41598_2021_85701_MOESM1_ESM.pdf]

Supplementary Data

Solvent Assisted Evolution and Growth Mechanism of Zero to Three Dimensional ZnO Nanostructures for Dye Sensitized Solar Cell Applications

Ramya M[a], Nideep T K[a], Nampoori V P N[a], Kailasnath M[*a]
[a]International School of Photonics, Cochin University of Science and Technology, Kochi, India.
e-mail: kailas@cusat.ac.in

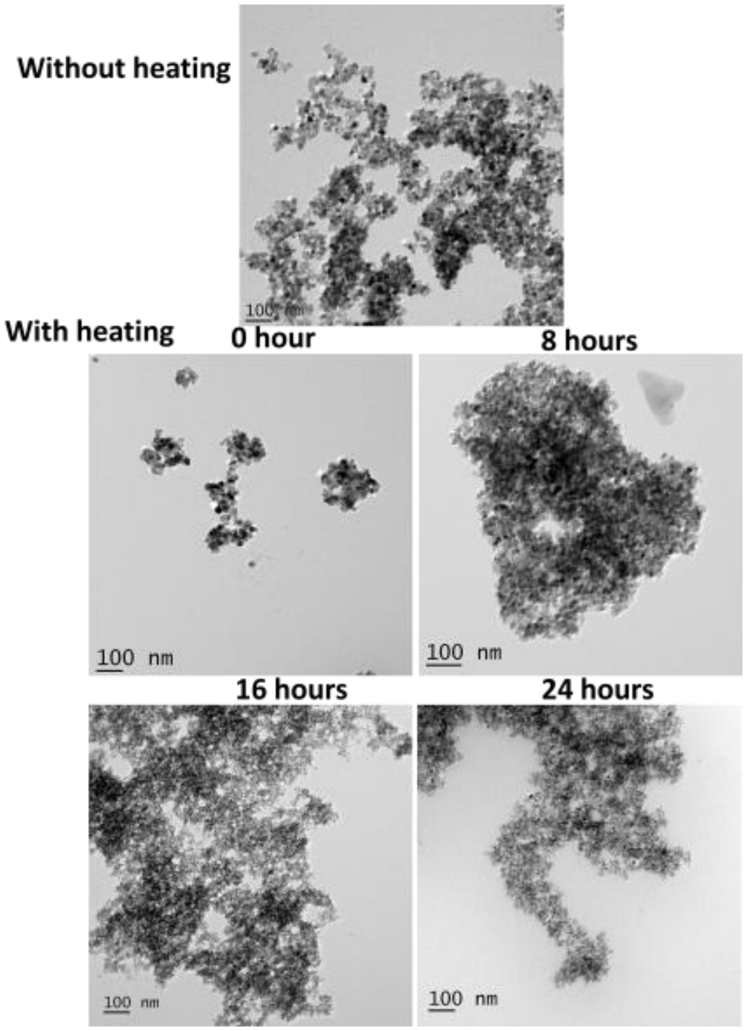

Figure S1: TEM image of ZnO nanodot in $C_2H_6O_2$ without and with heating.

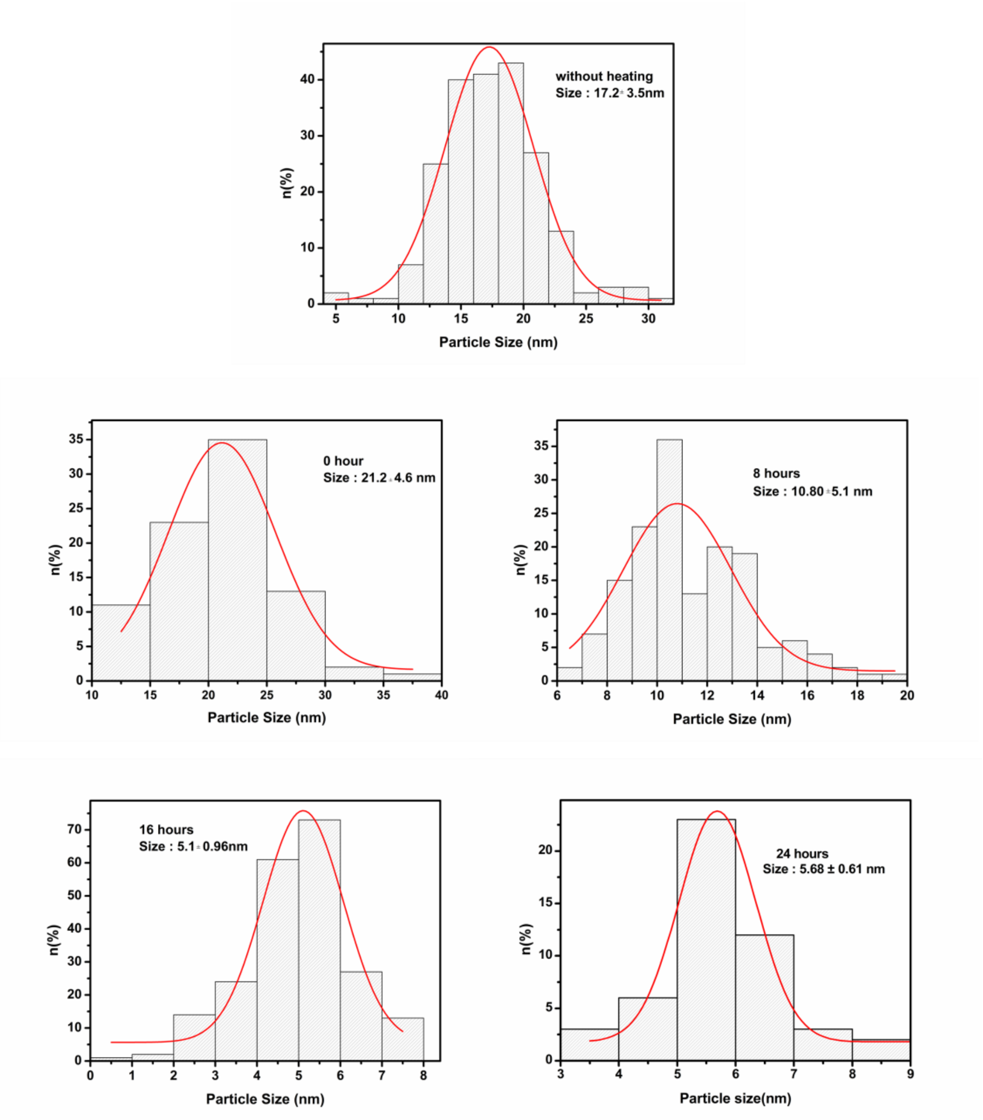

Figure S2: Size distribution of ZnO nanodot in $C_2H_6O_2$ as solvent.

Experimental procedures are repeated for understanding the effect of various parameters such as pH (pH 8, pH 9, pH 12), reaction temperature (80℃, 100℃, 120℃, and 150℃), reaction time (2hours, and 24hours) and surfactants (Triton X-100, CTAB, and PVP).

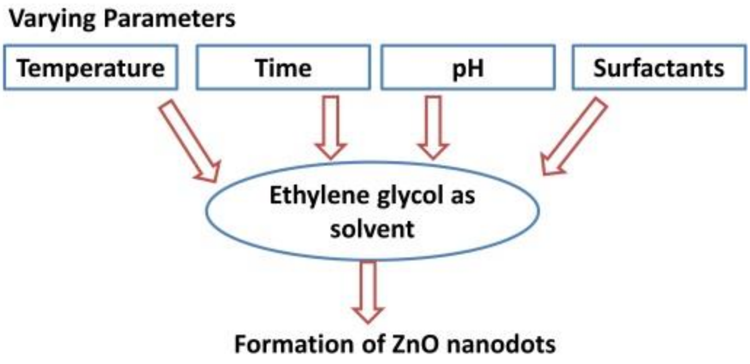

Figure S3: Varying reaction parameters in formation of ZnO nanodot using $C_2H_6O_2$ as solvent.

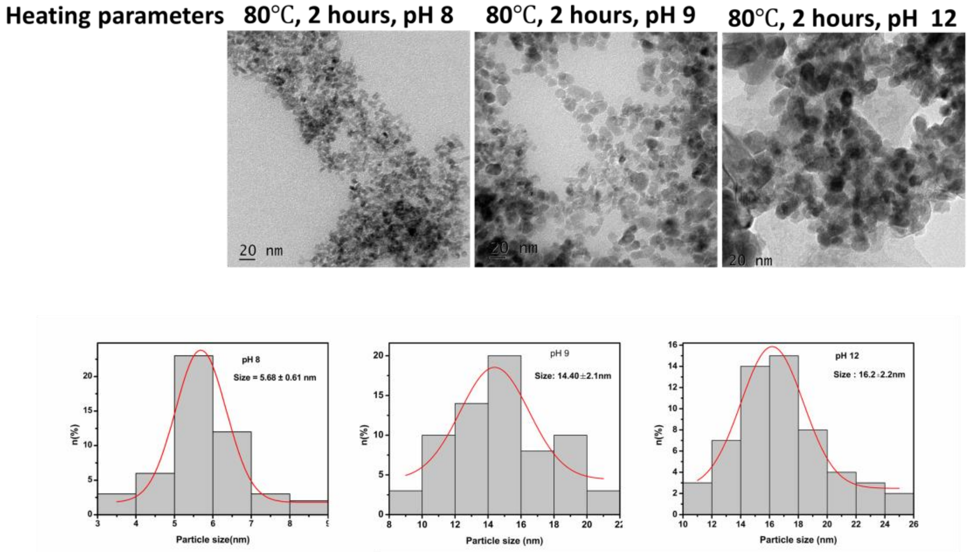

Figure S4: TEM image and Size distribution of ZnO nanodot in $C_2H_6O_2$ in various pH.

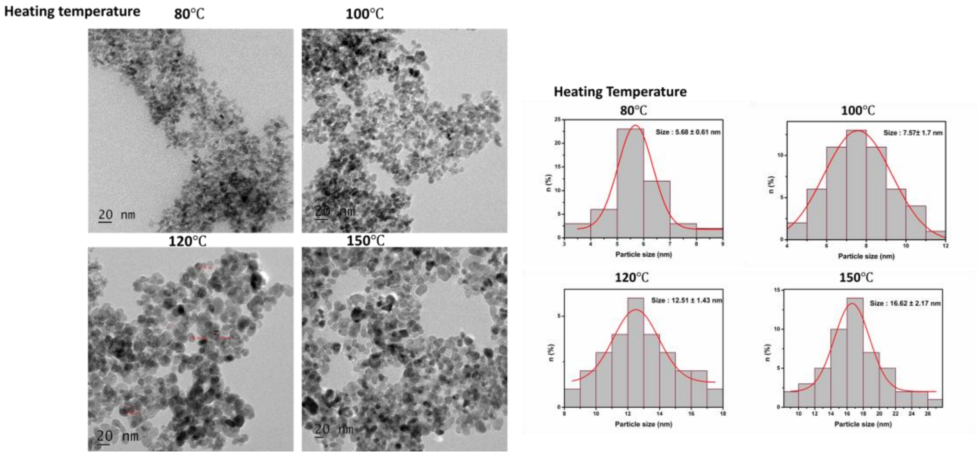

Figure S5: TEM image and Size distribution of ZnO nanodot in $C_2H_6O_2$ in various reaction temperatures.

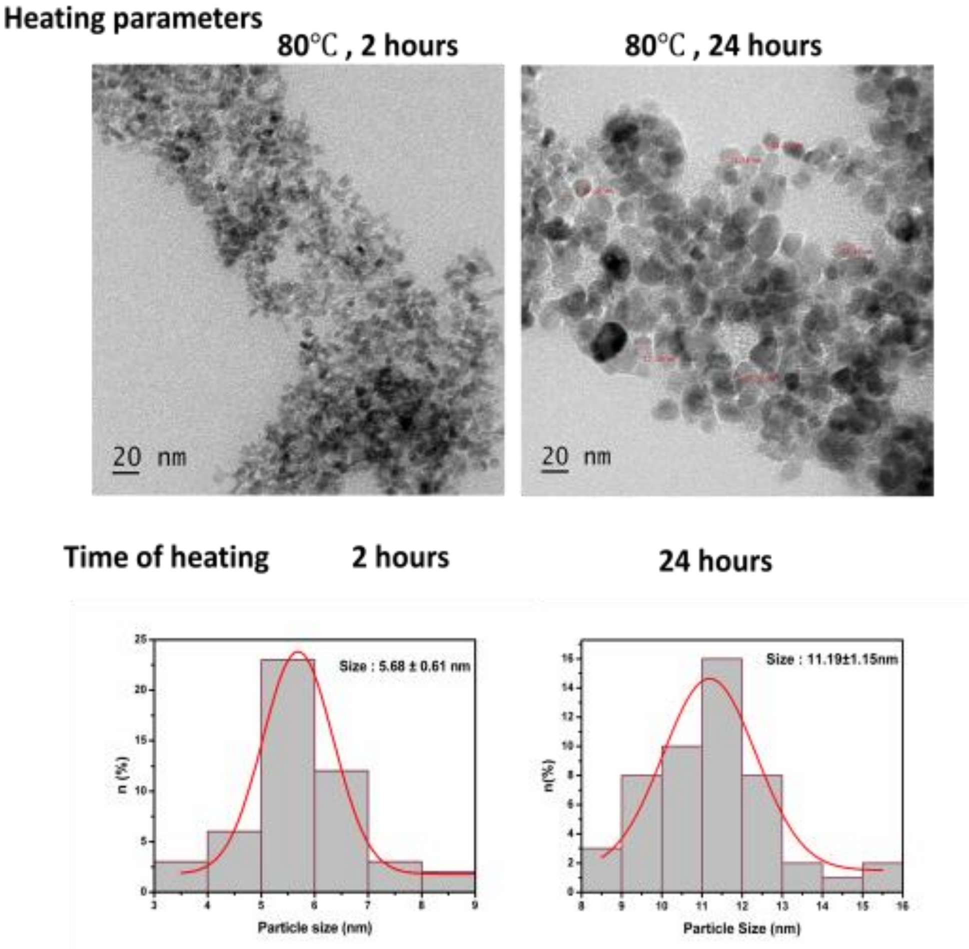

Figure S6: TEM image and Size distribution of ZnO nanodots in $C_2H_6O_2$ in various reaction times.

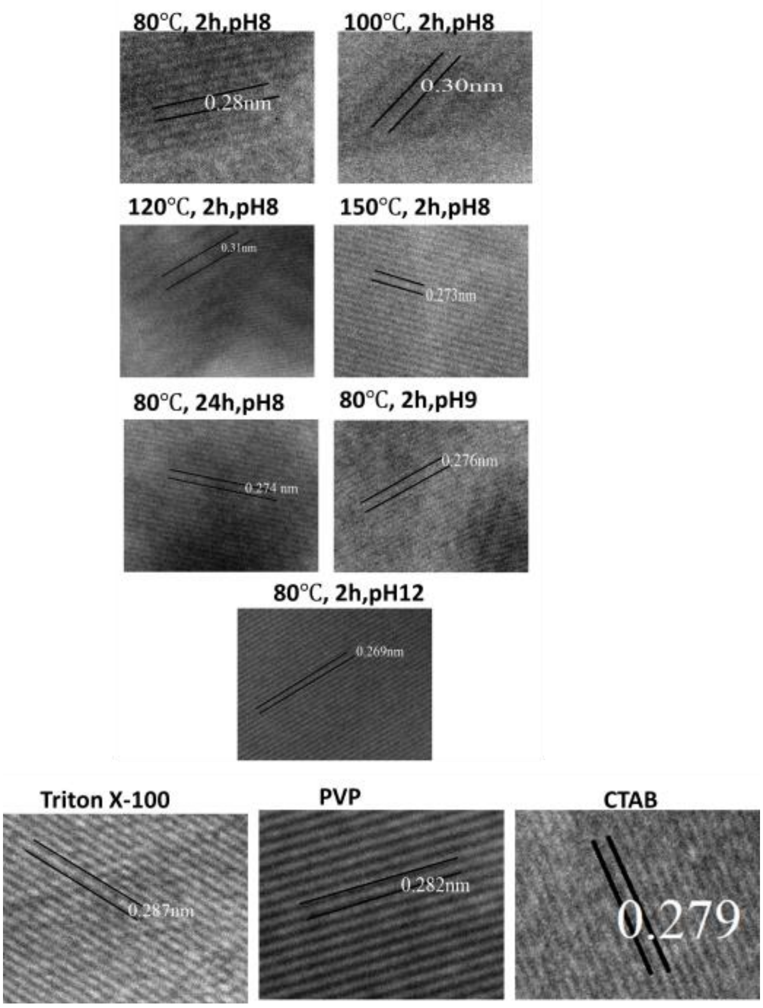

Figure S7: HRTEM image of ZnO nanodots in $C_2H_6O_2$ as solvent.

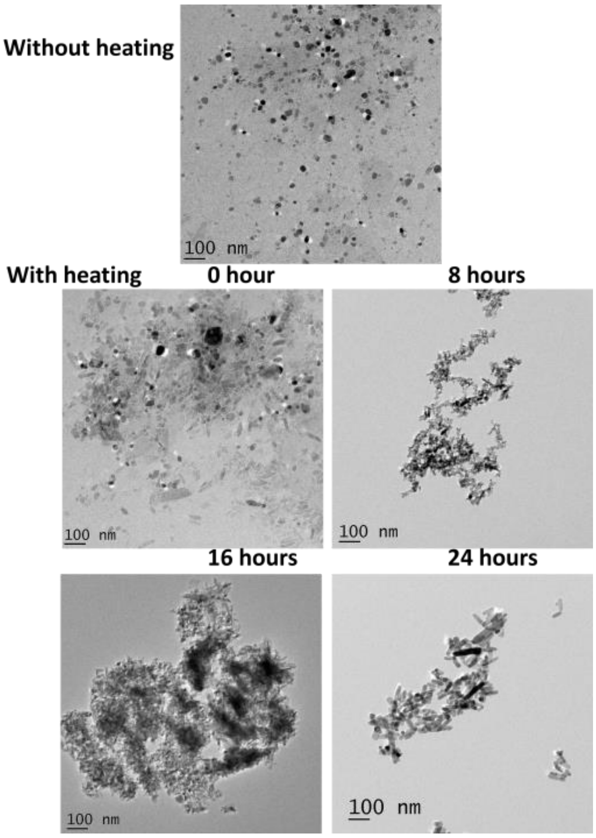

Figure S8: TEM image of ZnO nanostructure in $C_4H_{10}O$ without and with heating.

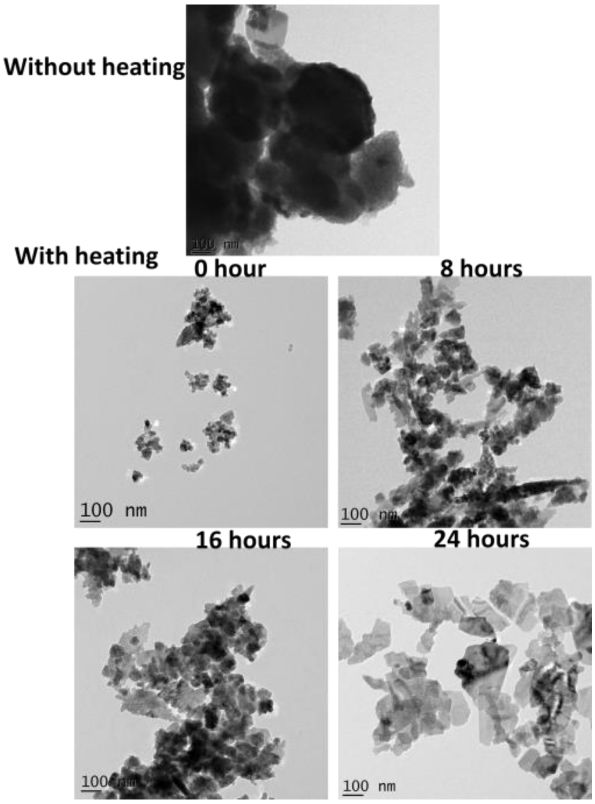

Figure S9: TEM image of ZnO nanostructure in CH_3COOH without and with heating.

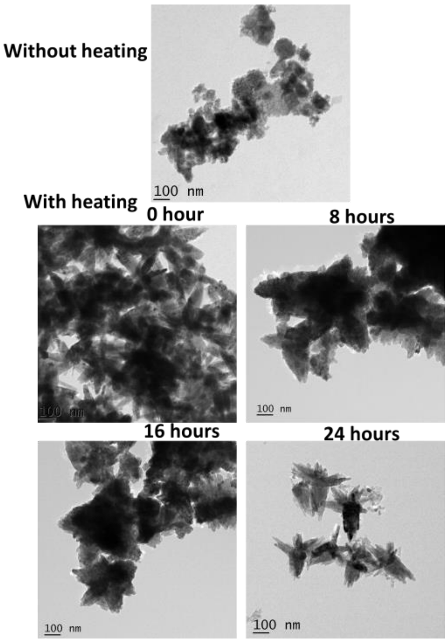

Figure S10: TEM image of ZnO nanostructure in H_2O without and with heating.

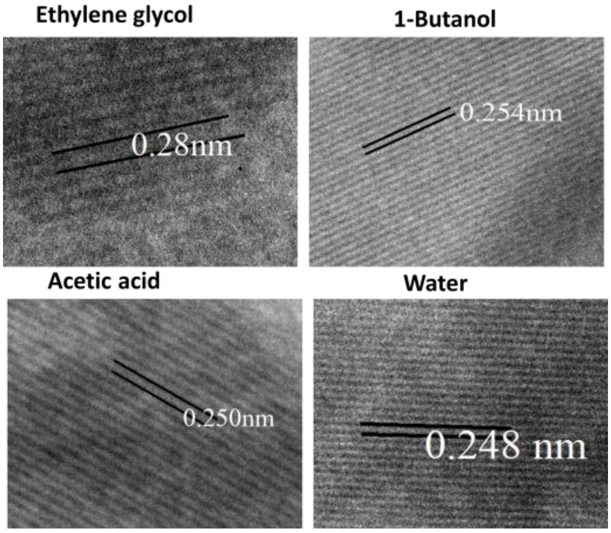

Figure S11: HRTEM image of synthesized ZnO nanostructures in distinct solvents after 1 day aging.

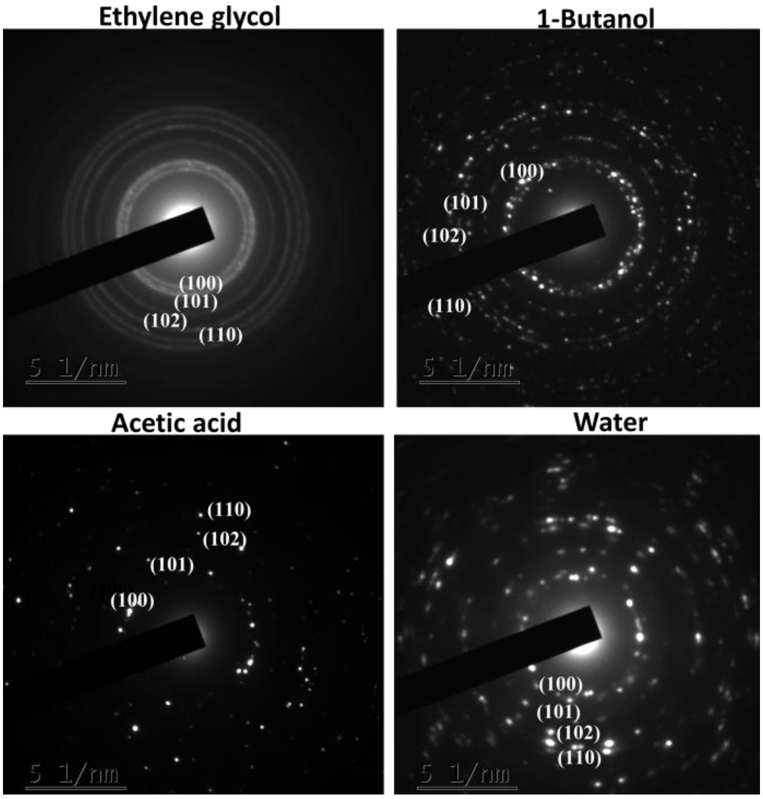

Figure S12: SAED pattern of synthesized ZnO nanostructures in distinct solvents after 1 day aging.

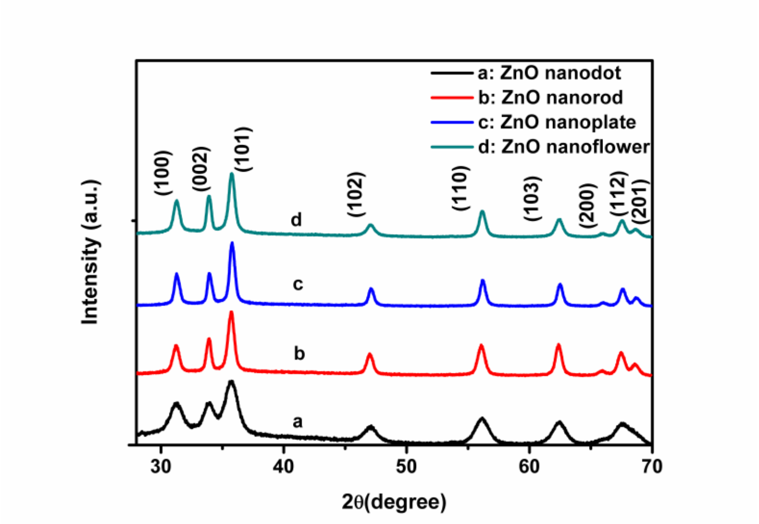

Figure S13: XRD pattern of synthesized ZnO nanostructures in distinct solvents.

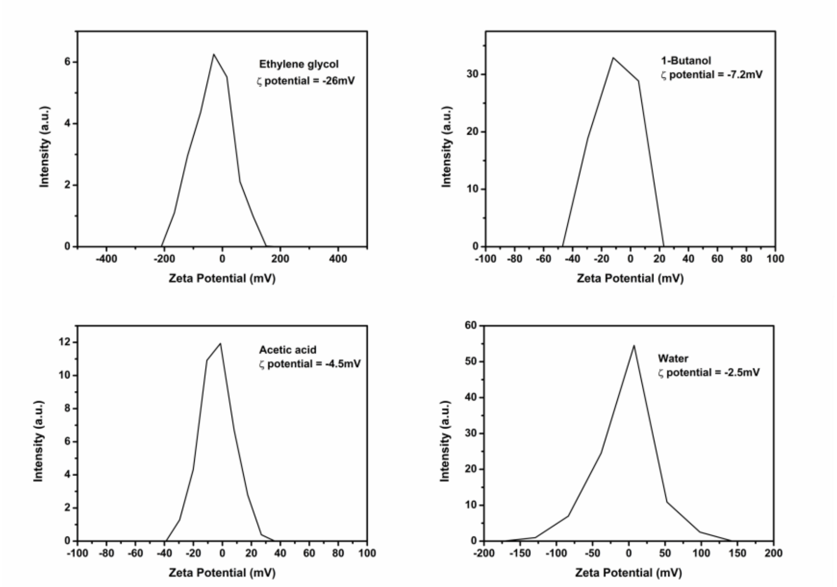

Figure S14: Zeta potential value of ZnO nanostructures in distinct solvents.

Table S1: Physicochemical properties and zeta potential value of used solvents in synthesis of ZnO nanostructures.

Solvents	Morphology	Relative Polarity	Dielectric Constant	Zeta Potential (mV)
$C_2H_6O_2$	Nanodot	0.790	38	-26
$C_4H_{10}O$	Nanorod	0.586	17.5	-7.2
CH_3COOH	Nanoplate	0.648	6.1	-4.5
H_2O	Nanoflower	1	80	-2.5

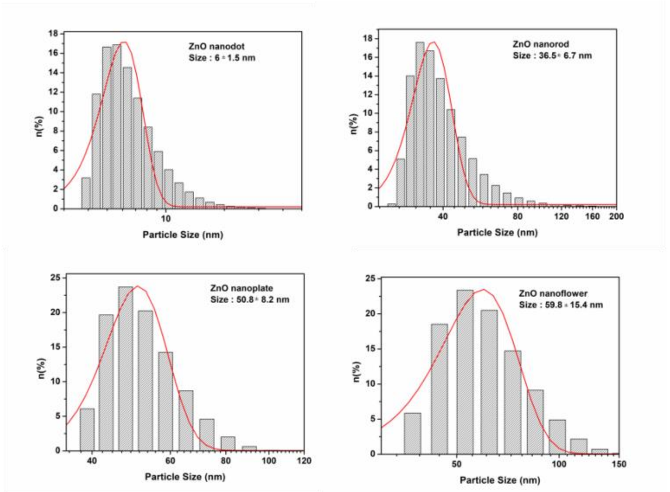

Figure S15: Particle size distribution of ZnO nanostructures in distinct solvents using DLS particle size analyser

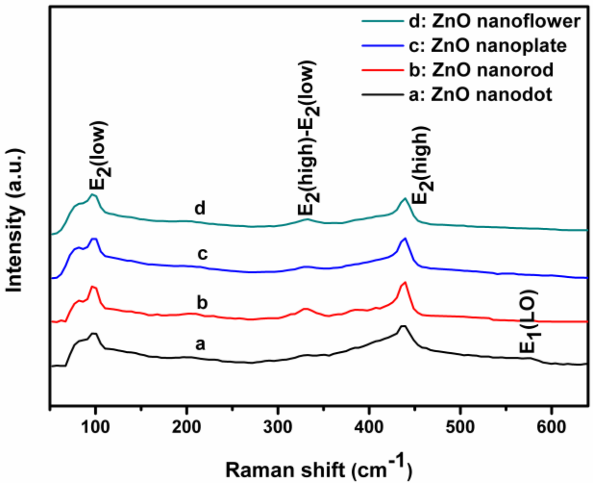

Figure S16: Raman spectra of ZnO nanostructures in distinct solvents.

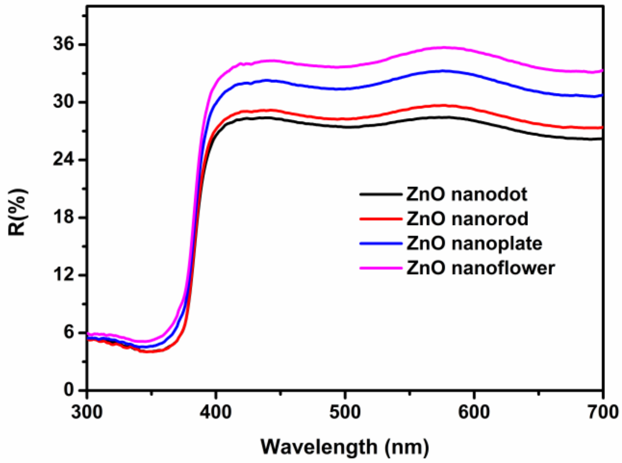

Figure S17: Reflectance spectra of ZnO nanostructure films.

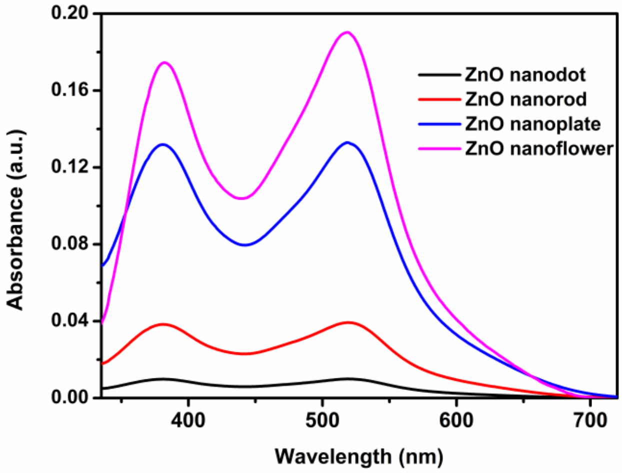

Figure S18: UV-vis absorbance spectra of solution containing dye desorbed from the sensitized photoanodes.
